# Changes in leisure-time physical activity after transition to retirement: a follow-up study

**DOI:** 10.1186/1479-5868-8-36

**Published:** 2011-04-23

**Authors:** Jouni Lahti, Mikko Laaksonen, Eero Lahelma, Ossi Rahkonen

**Affiliations:** 1 Department of Public Health, University of Helsinki, Helsinki, Finland

**Keywords:** Physical activity, retirement, health, follow-up

## Abstract

**Background:**

Retirement is a major life change that is likely to affect lifestyles. The aim of this study was to examine changes in leisure-time physical activity of moderate and vigorous intensity among ageing employees facing transition to retirement over a follow-up of 5-7 years.

**Methods:**

The baseline data were collected by questionnaire surveys in 2000-2002 among 40-60-year-old employees of the City of Helsinki. A follow-up survey was conducted among the baseline respondents in 2007 (n = 7332, response rate 83%). Those who were on disability retirement at the follow-up were distinguished from old-age retirees. Leisure-time physical activity was measured using similar questions in both surveys.

**Results:**

Old-age retirees increased significantly their time spent in moderate-intensity physical activity: women 31 minutes per week and men 42 minutes per week on average. Such changes were not found among disability retirees or those remaining employed. There were no changes in vigorous activity. Leisure-time physical inactivity at follow-up was lower among old-age retirees compared with employees of nearly the same age. Adjustments made for potential baseline covariates had no effects on these findings.

**Conclusions:**

Transition to old-age retirement was associated with an increase in moderate-intensity leisure-time physical activity and a decrease in the proportion of inactive. Encouraging people to leisure-time physical activity after retirement is worthwhile as the increase in free time brings new possibilities for it.

## Background

Engaging in physical activity is an important part of healthy ageing [1]. Lack of physical activity is associated with the risk of chronic diseases and loss of functioning as well as poor quality of life [2]. However, many older adults fail to reach the minimum amount of physical activity recommended for maintaining good health [3,4]. The population is ageing in most Western countries and in Finland even more rapidly than elsewhere [5] thereby making physical activity among older adults an increasingly important public health issue.

Due to the ageing populations ever larger numbers of people are facing retirement, a major life change with possible consequences for lifestyles. Physical activity typically declines as people are ageing [6] and after retirement the work related physical activity is lost. However, retirement also brings opportunities for increasing physical activity as the time previously spent for work can be spent on leisure-time activities. Working age people often mention lack of time as a reason for not participating in leisure-time physical activities [7]. Also other factors besides time related barriers, such as changes in social networks and concerns about health and independence later in life [8], may affect leisure-time physical activity near the retirement age.

The formal retirement age in Finland varies from 63 to 68 years. However, early retirement due to disability has become more common and the true average age of retirement currently is about 60 years [9]. It is important to distinguish different routes of retirement as disability that is a ground for early retirement often restricts participation in physical activity. Ill-health needs to be taken into account when physical activity among ageing people is examined, and longitudinal study design is required to capture the influence of retirement on physical activity.

There are only few prospective studies examining the changes in physical activity after transition to retirement [10-14]. A 6-year follow-up study conducted in the United States [10] concluded that those not in continuous work at the follow-up increased their leisure-time physical activity. A Scottish study [11] distinguished paid employment from no employment during a 4-5-year follow-up and concluded that among the 699 respondents the majority did too little physical activity outside work to compensate for the loss of physical activity at work. A Dutch study with 13-years follow-up [12] concluded that among the 971 respondents retirement from work reduced work-related transportation that was not compensated by increased leisure-time physical activity. Another study from the US [13] combined sports and physical labour in the measure of physical activity and found that physical activity increased with retirement from a sedentary job and that in the total sample retirement was not associated with increased physical activity. A 3-years follow-up from France [14] showed that retirement was associated with a two hour increase per week in moderate-intensity leisure-time physical activity. However, in these previous studies different retirement routes have not been distinguished and age differences between the retired and those remaining employed needs to be adequately considered.

The aim of this study was to investigate whether transition to old-age or disability retirement affects leisure-time physical activity. We examine the time spent in physical activities of moderate and vigorous intensity and also examine the proportion of those who are physically inactive. A further aim was to study whether baseline determinants such as body mass index, smoking, physical strenuousness of work, socioeconomic position and limiting longstanding illness explain differences in the change in leisure-time physical activity between those retiring and those remaining employed.

## Methods

The baseline data were derived from the Helsinki Health Study questionnaire surveys in 2000, 2001 and 2002 among employees of the City of Helsinki who reached the ages of 40, 45, 50, 55 and 60 years during each survey year [15]. The sample at the baseline consisted of 13 346 persons of whom 67% returned the questionnaire (7148 women and 1799 men). The baseline data satisfactorily represent the target population, according to our non-response analyses [16]. A follow-up survey was sent to all baseline respondents in 2007 (n = 7332, response rate 83%). Respondents with missing information in any of the study variables (n = 432) and respondents non-employed (e.g. unemployed) at follow-up for other reasons than retirement (n = 194) were excluded from the present study. The analyses included 5453 women and 1253 men. The study was approved by the ethics committees of the Department of Public Health, University of Helsinki and the health authorities of the City of Helsinki.

### Employment status

Employment status, date of retirement and type of retirement pension was asked at the follow-up. Those who were on disability retirement (disability and individual early retirement pensioners, n = 231) were distinguished from other full-time retirees (e.g. old-age, early old-age and unemployment pensioners, n = 1057). Those who were on part-time retirement and working part-time were considered to be still in employment. Due to the nature of our physical activity measure (12 months recall) those who had retired within the previous six months were considered to be still in employment. The baseline sample included all employees who reached 40, 45, 50, 55 or 60 years in 2000, 2001 and 2002. For the analysis the participants were collapsed into groups of 40-50-year-olds and 55-60-year-olds.

### Leisure-time physical activity

Physical activity was measured identically at the baseline and the follow-up. The respondents were asked their average weekly hours of leisure-time physical activity/exercise (including commuting) within the previous 12 months in walking or other activities that correspond to the same intensity. A similar question was then presented for brisk walking, jogging, and running, or their equivalent activities. The respondents were asked to estimate how many hours per week they on average spent in physical activity corresponding to each grade of intensity. Each intensity grade had five response alternatives: no activity, 0-½ hours/week, ½-1 hours/week, 2-3 hours/week, and ≥ 4 hours/week. Class mid-points were used for the calculation of time spent in physical activity, e.g. 2-3 hours/week was substituted with 150 min/week. For each respondent the time spent in moderate-intensity physical activity (walking, and brisk walking, or their equivalent activities) and vigorous physical activity (jogging, and running, or their equivalent activities) were calculated separately. The total time spent in physical activity for the all four intensity grades were also calculated. The volume of leisure-time physical activity was assessed by approximate MET-minutes per week [17] and calculated by multiplying the time spent by the estimated MET value of each physical activity grade [18], and then adding the four values together. In addition to the time spent in physical activity also inactivity was analysed. Based on physical activity recommendations the respondents were classified as inactive if the volume of physical activity was under 840 MET-minutes per week [19]. The Finnish physical activity guidelines [20] recommend brisk walking for 30 min on five days per week or equivalent volume of at least moderate-intensity activity. For those retiring during the follow-up the physical activity measure included leisure-time activities and for those still working commuting was also included.

### Covariates measured at the baseline

Body mass index (BMI) was calculated by using the baseline questionnaire data on height and weight. Smoking status was classified into two groups: non-smokers and smokers. The respondents were asked how physically strenuous their work was and those reporting physically strenuous work were separated from others. Socioeconomic position (SEP) was classified into four occupational social classes: managers and professionals, semi-professionals, routine non-manual, and manual workers [15]. Limiting longstanding illness (LLI) was classified into two groups: LLI and no LLI.

### Statistical methods

Analyses were conducted separately for women and men. Proportions and means with standard deviations (SD) of the baseline study variables by employment status at the follow-up were calculated for descriptive purposes. The time spent in leisure-time physical activity and its mean change including 95% confidence intervals (CI) was calculated for the total time and separately for moderate-intensity and vigorous physical activity by employment status at the follow-up. The GLM procedure was used to calculate adjusted means and 95% confidence intervals (CI) for change in the total time spent in physical activity by employment status. The GENMOD procedure was used to calculate adjusted odds ratios (OR) for physical inactivity at the follow-up to compare old-age retirees and employees 55-60-year-old. In order to obtain an age group as comparable as possible to the retired those still employed at the follow-up were divided into two age groups. SAS version 8.02 for Windows was used for the analyses (SAS Institute, Chicago, IL, USA).

## Results

Table 1 shows the distribution of the baseline study variables by employment status at the follow-up. At the follow-up 15% of women and 20% of men were retired. Three to four percent were on disability retirement. Differences in BMI between employment status groups were small, women on disability retirement, however, had a high BMI. Old-age retirees were less often smokers than employees, whereas disability retirees were most often smokers at the baseline. Physically strenuous work was prevalent among disability retirees. Among the 55-60-year-old male employees physically strenuous work was not as prevalent as among old-age retirees, whereas among women such differences were not found. Physically strenuous work was more prevalent among women than among men. Those on disability retirement also tended to come from lower SEP groups, especially among women. LLI was highly prevalent at the baseline among those on disability retirement at the follow-up and also slightly more prevalent among old-age retirees than among employees. The prevalence of physical inactivity at the baseline slightly differed between employees and retirees, especially among women. There were also differences in the time spent in physical activity at the baseline although not between old-age retirees and 55-60-year-old employees.

**Table 1:** Description of the baseline study variables by employment status at the follow-up among women and men

	Employees aged 40-50 y	Employees aged 55-60 y	Old-age retirees	Disability retirees	All
**Women (n)**	**3398**	**1066**	**802**	**187**	**5453**
Age years (SD)	45 (4.1)	55 (1.1)	58 (2.5)	54 (4.6)	49 (6.6)
BMI (SD)	25 (4.2)	26 (4.4)	26 (4.1)	28 (6.2)	25 (4.4)
Smokers (%)	24	19	12	29	22
Physical work (%)	38	38	40	59	39
SEP (%)					
Manual	11	12	10	21	11
Routine non-manual	41	42	39	56	42
Semi-professionals	22	15	18	12	19
Managers/professionals	27	30	34	11	28
LLI ^a ^(%)	13	19	24	58	18
Minutes PA/week (SD)	317 (200)	293 (186)	295 (194)	275 (204)	308 (197)
Inactive ^b ^(%)	22	26	29	35	24
**Men (n)**	**672**	**282**	**255**	**44**	**1253**
Age years (SD)	45 (4.1)	55 (1.2)	58 (2.4)	55 (3.7)	51 (6.6)
BMI (SD)	26 (3.8)	26 (4.1)	27 (3.9)	27 (3.6)	26 (3.9)
Smokers (%)	27	22	20	39	25
Physical work (%)	18	6	14	23	15
SEP (%)					
Manual	25	16	28	30	24
Routine non-manual	12	7	6	18	10
Semi-professionals	20	20	18	16	19
Managers/professionals	43	57	47	36	47
LLI ^a ^(%)	14	12	22	50	16
Minutes PA/week (SD)	323 (230)	305 (212)	302 (206)	275 (164)	313 (220)
Inactive ^b ^(%)	24	26	27	25	25

^a^Those with limiting longstanding illness (LLI)

^b ^Under 840 MET-min/week

In all age groups, changes in physical activity were small among those still employed (Table 2). However, old-age retirees increased markedly their time spent for moderate-intensity leisure-time physical activity. Mean increase among women was 31 (95% CI = 18-44) min per week and among men 42 (95% CI = 18-67) min per week. Changes in vigorous activity were not observed among old-age retirees. Among women on disability retirement there was a slight decrease in overall time spent in leisure-time physical activity but among men a slight increase was seen.

**Table 2:** Change from the baseline (min per week (95% CI)) and time spent (min per week) in physical activity (total, moderate, vigorous) at the follow-up by employment status and by age groups of those still employed among women and men

	n	Change total	Time total	Change moderate	Time moderate	Change vigorous	Time vigorous
**Women**	5453						
Employees		7 (1-13)	318 (312-324)	2 (-4-7)	278 (273-283)	5 (3-8)	40 (38-43)
40	1095	9 (-4-21)	329 (317-340)	-4 (-16-7)	269 (259-279)	13 (7-19)	59 (53-65)
45	1132	7 (-5-20)	320 (309-332)	1 (-10-13)	276 (266-286)	6 (0-12)	45 (39-50)
50	1171	1 (-12-13)	318 (307-330)	-1 (-12-10)	284 (274-294)	2 (-4-7)	35 (29-40)
55-60	1066	11 (-2-24)	304 (293-316)	11 (-1-22)	281 (271-291)	0 (-5-6)	23 (18-28)
Old-age retirees	802	29 (15-44)	324 (310-338)	31 (18-44)	304 (292-315)	-2 (-8-4)	20 (15-26)
Disability retirees	187	-7 (-37-23)	268 (239-296)	-3 (-30-24)	257 (232-281)	-4 (-17-9)	11 (0-24)
**Men**	1253						
Employees		0 (-15-15)	318 (304-332)	8 (-5-21)	239 (228-251)	-7 (-15-0)	79 (71-88)
40	204	-7 (-39--26)	342 (310-373)	3 (-25-30)	223 (198-248)	-9 (-27-8)	119 (100-137)
45	226	4 (-27-35)	315 (285-345)	-6 (-32-20)	227 (204-250)	10 (-6-27)	88 (70-106)
50	242	-6 (-36-24)	308 (278-337)	6 (-20-31)	245 (222-267)	-11 (-27-5)	63 (47-81)
55-60	282	8 (-20-36)	313 (286-339)	25 (2-48)	256 (235-277)	-17 (-32-(-2))	57 (42-72)
Old-age retirees	255	42 (13-71)	344 (316-371)	42 (18-67)	291 (269-313)	-0 (-15-15)	52 (37-68)
Disability retirees	44	16 (-54-85)	291 (225-357)	19 (-39-78)	264 (211-318)	-4 (-40-33)	27 (0-69)

Among those still employed changes in physical activity were minor (Table 2), but differences in the time spent between age groups were found. The differences between age groups were pronounced in vigorous physical activity as the 40-year-old employees spent double the time in vigorous activities compared to the 55-60-year-old employees among both genders. Old-age retirees spent as much time in the total leisure-time physical activity as did the 40-year-old employees. Disability retirees spent the least time in leisure-time physical activity. Especially, the time spent in vigorous activity was very low among disability retirees. Men spent more time in vigorous and less time in moderate activity than women regardless of age and employment status, although gender differences in moderate activity were minor among older employees and old-age retirees.

We then examined whether adjustments for baseline BMI, smoking, physical strenuousness of work, SEP and LLI contributed to the differences in the change of time spent in total leisure-time physical activity between retiree groups and employees (Table 3). These adjustments, however, had no effects on the differences found between employees and old-age retirees. We also tested for interactions to examine whether the effect of old-age retirement on change in physical activity differed by baseline BMI, smoking, physical strenuousness of work, SEP and LLI. This was done by comparing old-age retirees to 55-60-year-old employees. No statistically significant interactions were found.

**Table 3:** Change in time spent in physical activity (min per week (95% CI)) by employment status adjusted for smoking and BMI, physical work, SEP, LLI among women and men

	Unadjusted	Model 1	Model 2	Model 3	Model 4
**Women**					
Employees 40-50	6 (-2-13)	6 (-2-13)	6 (-2-12)	5 (-2-13)	6 (-1-13)
Employees 55-60	11 (-2-24)	11 (-2-24)	11 (-2-24)	11 (-1-24)	11 (-2-24)
Old-age retirees	29 (15-44)	28 (14-43)	30 (15-44)	27 (13-42)	29 (14-44)
Disability retirees	-7 (-37-23)	-5 (-35-26)	-3 (-33-27)	1 (-29-32)	-9 (-40-22)
**Men**					
Employees 40-50	-3 (-20-15)	-2 (-20-16)	-2 (-20-16)	-1 (-19-16)	-2 (-20-16)
Employees 55-60	8 (-19-35)	7 (-20-35)	7 (-21-34)	3 (-25-31)	9 (-19-37)
Old-age retirees	42 (13-71)	41 (12-70)	42 (13-71)	43 (14-72)	40 (13-71)
Disability retirees	16 (-54-85)	15 (-55-85)	17 (-53-87)	21 (-49-90)	5 (-66-76)

Model 1 adjusted for body mass index (BMI) and smoking

Model 2 adjusted for physical work

Model 3 adjusted for socioeconomic position (SEP)

Model 4 adjusted for limiting longstanding illness (LLI)

Lastly we compared leisure-time physical inactivity at the follow-up between old-age retirees and 55-60-year-old employees (Table 4). After adjusting for baseline physical inactivity the old-age retirees showed less physical inactivity among women (OR = 0.78, 95% CI: 0.62, 0.99). Among men the odds ratio was at a similar level (OR = 0.76, 95% CI = 0.50-1.15), however, statistically non-significant due to fewer men than women in the data. Adjustments made for other baseline covariates had no effects among women, while among men adjustments made for BMI, smoking, physical strenuousness of work, SEP and LLI slightly strengthened the difference found, which remained statistically non-significant (OR = 0.66, 95% CI: 0.43, 1.04). No interactions were found implying that the effect of old-age retirement on physical inactivity at the follow-up did not differ by baseline BMI, smoking, physical strenuousness of work, SEP and LLI.

**Table 4:** OR for physical inactivity at the follow-up (95% CI): old-age retirees compared to employees (55-60) adjusted for baseline inactivity, BMI and smoking, physical work, SEP, LLI among women and men

	Model 1	Model 2	Model 3	Model 4	Model 5
**Women**					
Employees 55-60	1.00	1.00	1.00	1.00	1.00
Old-age retirees	0.78 (0.62-0.99)	0.78 (0.62-0.99)	0.77 (0.61-0.99)	0.79 (0.62-1.01)	0.77 (0.60-0.99)
**Men**					
Employees 55-60	1.00	1.00	1.00	1.00	1.00
Old-age retirees	0.76 (0.50-1.15)	0.74 (0.48-1.13)	0.71 (0.46-1.10)	0.69 (0.44-1.07)	0.66 (0.43-1.04)

Model 1 baseline inactivity adjusted

Model 2 1+body mass index (BMI) and smoking

Model 3 2+ physical work

Model 4 3+ socioeconomic position (SEP)

Model 5 4+ limiting longstanding illness (LLI)

## Discussion

The main aim of this study was to examine whether retirement influences leisure-time physical activity of moderate and vigorous intensity. We found that old-age retirees increased their time for moderate-intensity leisure-time physical activity as the mean increase among women was 31 minutes per week and among men 42 minutes per week. Among 40-50-year-old and 55-60-year-old employees and disability retirees the time spent for physical activity did not change from the baseline. The time spent in vigorous physical activity at the follow-up showed a gradual decrease from 40-year-old to 55-60-year-old employees and further among old-age retirees, while moderate activity was more common among 55-60-year-old employees and old-age retirees. Adjustments made for baseline covariates did not explain the differences between retirees and employees. The occurrence of physical inactivity at the follow-up was lower among old-age retirees compared with older employees of nearly the same age.

The results from previous prospective studies are inconclusive and direct comparisons are not warranted due to different study settings and measures of physical activity. Some have measured leisure-time physical activity [10,12,14] similarly to our study, while others have used a broader approach to measuring physical activity [11,13]. In one study the measures of physical activity varied between measurement points [12]. Moderate activity was not separated from vigorous activity [11] and one study measured only vigorous activity [13]. Our study showed that the increase in leisure-time physical activity associated with retirement is due to increases in moderate activity such as walking. These findings are similar to previous studies that have considered the intensity of physical activity [10,14]. A French study [14], however, used a highly selected group of intervention study participants in their study with complete data on physical activity and working status available only on every fourth subject. This selection of active participants might lie behind the substantial two hours weekly increase found in their study. Study settings have also been different with follow-up time varying from three to thirteen years. The definition of retirement has been vague in many previous studies [10,11,13] and none have distinguished between retirement due to disability and old-age. Our study showed that those retired due to disability did not markedly change their leisure-time physical activity. Furthermore, since the old-age retirees are necessarily older than those remaining in employment the effect of retirement is difficult to separate from the possible effect of age. We found modest differences in the changes of the time spent for physical activity between age groups among those remaining in employment. Thus there was no general tendency of physical activity to increase by age, but among those retiring due to old-age there was a clear step up in physical activity. This supports the effect of retirement rather than age on physical activity.

In this study moderate activity was common among the 55-60-year-old employees and old-age retirees, while vigorous activity was more common among the 40-50-year-old employees. Among those on disability retirement participation in vigorous physical activity was very low. These were expectable observations as age and health are associated with the intensity of physical activity [6]. It should be noted that we examined the absolute intensity of physical activity. Moderate activity such as brisk walking may be as strenuous for example for a 65 year old as jogging for a 45 year old person. Also other characteristics besides age, such as weight and aerobic fitness, determine how much energy is expended in a given activity.

The mean increase in leisure-time physical activity among old-age retirees found in this study is relatively small considering that almost 40 hours of working time per week can be spent for leisure after retirement. This study examined leisure-time physical activity and changes in overall physical activity were of secondary interest. We adjusted for physical strenuousness of work but it had no effects on the differences found between old-age retirees and employees. Adjusting for other baseline covariates neither explained the differences found. In addition we checked that the effect of old-age retirement on physical activity did not differ by physical strenuousness of work or other baseline covariates. This might be due to retirement from work being an independent event during the life-course affecting positively the time people have for leisure pursuits and the baseline covariates are simply unlikely to contribute to these differences found. However, evidence from previous studies suggest the importance of physical work for physical activity after retirement [11,13].

Leisure-time physical inactivity decreased during follow-up among old-age retirees but not among older employees of nearly the same age. This provides further evidence that leisure-time physical activity increases after transition to old-age retirement. In addition this suggests that the increase in physical activity is prevalent among those classified as physically inactive at the baseline. It should be noted that even modest increases during the follow-up especially in vigorous activity may decrease the proportion of the inactive as at the baseline they may have been somewhat, although not sufficiently, active (0-840 MET-min/week). At the baseline around a quarter of all participants were classified as physically inactive and inactivity was slightly more common among those who retired during follow-up than among those who remained employed. We were unable to examine changes in sedentary behaviour in addition to leisure-time physical inactivity. Previous studies, however, have shown that sedentary behaviour such as television viewing tends to increase after transition to retirement [11,14]. Nevertheless, avoiding physical inactivity is highly important for people near retirement age for several reasons. A recent study concluded that among middle-aged men increased physical activity was eventually associated with a reduction in mortality comparable to smoking cessation [21]. Another study concluded that among middle-aged women increased physical activity was associated with improved quality of life [22]. Also our previous study showed the importance of leisure-time physical activity for daily functioning among the middle-aged [19] while the benefits of physical activity and fitness for functioning and independence among older people are also well known.

### Strengths and limitations

The strengths of our study include a relatively large and recent sample of women and men, and a prospective design with five to seven years between the baseline and the follow-up. Another strength is that leisure-time physical activity was measured identically at both the baseline and the follow up surveys. Further advantages include data providing information on employment status as well as date and type of retirement pension distinguishing disability from old-age retirement.

There are also limitations. Those reporting the maximum time of leisure-time physical activity, i.e. over four hours per week at the baseline cannot report more physical activity of that grade even if the time spent for it in fact had increased. This ceiling effect might be more pronounced in the older age groups that participate more in moderate-intensity activities and thus might somewhat underestimate the degree of positive change among the retired. Commuting physical activity was integrated in the question which prevents us to examine the contribution of commuting to the time spent in leisure physical activity among those remaining employed. The information on leisure-time physical activity was self-reported which is prone to overestimation although there is no reason to believe that this would differ between those retired and those employed at the follow-up. The type of retirement pension was determined at the follow-up survey. Some of the old-age retirees may have moved from disability to old-age pensions before the follow-up which might cause underestimation in the true positive impact of old-age retirement on physical activity. The disability retirees are also a diverse group with regard to the diseases leading to early retirement. While in general disability retirees spent least time in physical activity some of them may actually increase their activity e.g. as a part of rehabilitation. Attrition is a common problem in prospective studies. Our response rate was high at the follow-up, counteracting the bias due to attrition. The study was based on baseline survey and one follow-up measurement. Having more measurement points would have allowed better to separate the effect of retirement from the possible effect of age.

## Conclusions

Old-age retirement increases participation in moderate-intensity leisure-time physical activity and decreases the proportion of those not engaging in physical activity as recommended for promoting health and preventing disease. These results are encouraging from the public health perspective: at least some people increase their leisure-time physical activity after retirement and the prevalence of physical inactivity is relatively low. Even if people are inactive at working age it is not too late to start and increase physical activity after retirement. Encouraging people to leisure-time physical activity after retirement is worthwhile as the increased free time brings new possibilities for it.

## Abbreviations

BMI: body mass index; CI: confidence interval; LLI: limiting longstanding illness; OR: odds ratio; SEP: socioeconomic position

## Competing interests

The authors declare that they have no competing interests.

## Authors' contributions

JL performed statistical analyses, interpreted results and drafted the manuscript. JL, ML, EL and OR contributed to designing the study, interpreting results and drafting the manuscript. All authors critically reviewed the manuscript and approved the final version.
